# Cathelicidin-Derived Synthetic Peptide Improves Therapeutic Potential of Vancomycin Against *Pseudomonas aeruginosa*

**DOI:** 10.3389/fmicb.2019.02190

**Published:** 2019-09-19

**Authors:** Imran Mohammed, Dalia G. Said, Mario Nubile, Leonardo Mastropasqua, Harminder S. Dua

**Affiliations:** ^1^Academic Ophthalmology, Division of Clinical Neuroscience, School of Medicine, University of Nottingham, Nottingham, United Kingdom; ^2^Ophthalmology Clinic, University “G. d’Annunzio” of Chieti-Pescara, Chieti, Italy

**Keywords:** *Pseudomonas aeruginosa*, antimicrobial peptides, vancomycin, LL-37, FK13, FK16, antibiotic resistance, bacterial keratitis

## Abstract

*Pseudomonas aeruginosa* (PA) is the leading cause of corneal blindness worldwide. A constant increase in multi-drug resistant PA strains have heightened the challenge of effectively managing corneal infections with conventional antibiotics. Antimicrobial peptides are promising antibiotic analogs with a unique mode of action. Cathelicidin-derived shorter peptides (FK13 and FK16) have previously been shown to kill a range of pathogens in both *in vitro* and *in vivo* systems. Here, our aim was to exploit the potential of FK13 or FK16 to enhance the anti-*Pseudomonas* activity of vancomycin, which normally has low clinical efficacy against PA. Our results have demonstrated that FK16 is more potent than FK13 against different PA strains including a clinical isolate from a patient’s ocular surface. FK16 was shown to enhance the membrane permeability of PAO1 at sub-inhibitory concentrations. Moreover, FK16 at lower concentrations was shown to increase the antibacterial susceptibility of vancomycin against PA strains up to eightfold. The bactericidal synergism between FK16 and vancomycin was shown to be stable in the presence of physiological tear salt concentration and did not cause toxic effects on the human corneal epithelial cells and human red blood cells. Our results have revealed that sub-inhibitory concentration of FK16 could augment the antimicrobial effects of vancomycin against PA. It is anticipated that the future exploitation of the peptide design approach may enhance the effectiveness of FK16 and its application as an adjuvant to antibiotic therapy for the treatment of multi-drug resistant infections.

## Introduction

*Pseudomonas aeruginosa* is a ubiquitous Gram-negative bacterium, which causes opportunistic fatal infections in patients with compromised immunity (e.g., HIV, burn, cancer, and cystic fibrosis) (Poole, 2011). It is also the commonest cause of bacterial keratitis in extended-use contact lens wearers. *Pseudomonas* keratitis (PsK) is a rapidly progressing disease which frequently leads to irreversible corneal scar or melt (Hazlett, 2007). Due to its nature of the emergency, patients are mostly hospitalized requiring aggressive treatment with mono or fortified topical drops formulations (Otri et al., 2013). However, fortified drops have been shown to cause toxicity, tissue damage and reduce wound-healing (Dua et al., 2012; Austin et al., 2017). Severe PsK cases that require surgical transplantation of donor corneal tissue are often at potential risk of graft tissue rejection (Rush and Rush, 2016). Moreover, constant paucity of transplant tissue and recent emergence of multi-drug resistant (MDR) *P. aeruginosa* (MDRP) has further added to the challenge to manage PsK (Willcox, 2011; Morita et al., 2014; Vazirani et al., 2015; Tuli and Gray, 2016; Chojnacki et al., 2019).

In developing countries such as India and Brazil, ocular *P. aeruginosa* isolates have shown increased resistance to ciprofloxacin (25 to 54%), gentamicin (10 to 46%), and cefazolin/ceftazidime (80 to 97%) (Willcox, 2011). Moreover, 23% of cases of *Pseudomonas* keratitis at a single center in India were shown to be multi-drug resistant with very poor prognosis (Vazirani et al., 2015). In the last two decades, developed countries in North America and Europe have also reported an increased resistance of *P. aeruginosa* ocular isolates to first-generation cephalosporins, tobramycin, polymyxin B, and fluoroquinolones (Alexandrakis et al., 2000; Willcox, 2011; Asbell et al., 2018). Antimicrobial resistance poses a significant impact on public health and health services globally (Laxminarayan et al., 2013). One of the strategic guidelines of the World Health Organization (WHO) to combat antimicrobial resistance has been to promote judicial use of available antibiotics both in veterinary and hospital settings (Simonsen et al., 2004; Manyi-Loh et al., 2018). Another strategy has been to preserve the effectiveness of existing antimicrobials and the development of newer alternative therapies (O’Neill, 2016; Bloom et al., 2018).

Vancomycin, a tricyclic glycopeptide discovered in the 1950s, has greater specificity toward Gram-positive bacteria and widely used as a last resort treatment for methicillin-resistant *Staphylococcus aureus* (MRSA) infection (Alvarez et al., 2016; Yarlagadda et al., 2016b). Glycopeptide antibiotics are less preferred as monotherapy for the management of Gram-negative bacterial infections because of poor permeability through the outer membrane (Yarlagadda et al., 2016a). However, once this barrier is breached, for example with polymyxin antibiotic (colistin), vancomycin has shown efficacy against Gram-negative organisms. Clinical effectiveness of colistin-vancomycin combination against MDR Gram-negative infections has been previously demonstrated (Schina et al., 2006; Ceccarelli et al., 2015; O’Driscoll et al., 2018). Recent reports of colistin-resistant *P. aeruginosa* and potential nephrotoxicity of colistin have discouraged their further use (Poole, 2011; Poirel et al., 2017). This further highlights the clinical need for development and testing of alternative approaches for effective management of *P. aeruginosa* infections.

Antimicrobial peptides (AMPs) are naturally occurring host-defense molecules with unique microbicidal properties (Mohammed et al., 2017). These are considered to be a promising alternative to antibiotics for the treatment of MDR bacterial infections (Gordon et al., 2005). LL-37 is a lone member of the cathelicidin class of AMPs that is found in humans. It has been shown to display a broad-spectrum microbicidal activity against a range of pathogens (Mookherjee and Hancock, 2007). In addition, it also exhibits intracellular bactericidal and anti-biofilm activities against a variety of Gram-negative and Gram-positive bacteria (Wang et al., 2014; Luo et al., 2017). LL-37 is produced by a variety of immune and non-immune mammalian cells in response to different stimuli which subsequently aid in immunomodulation (Rosenfeld et al., 2006). However, despite its strong properties, the clinical use of LL-37 was limited due to its toxicity and cost of manufacturing. Recent studies have demonstrated that synthetic short peptides derived from LL-37 sequence such as KR12, FK13, and FK16 are capable of killing a variety of pathogens and do not elicit caustic immunologic responses and host tissue toxicity (Wang et al., 2012; Rajasekaran et al., 2017). Based on previous studies, we aimed to test whether these peptides are capable of enhancing the antimicrobial activity of vancomycin against different virulent strains of *P. aeruginosa* and assess the safety of peptide/antibiotic combination toward host cells.

First, we investigated the antibacterial efficacy of cathelicidin-derived shorter peptides and antibiotics in presence of the physiological salt concentration of tears, against a collection of *P. aeruginosa* strains including a clinical isolate from a patient with *Pseudomonas* keratitis. Next, we examined whether sub-inhibitory concentrations of FK13 or FK16 would increase the efficacy of vancomycin against *P. aeruginosa.* Finally, we assessed the cytotoxic effects of peptides and the combination of peptides and vancomycin on human corneal epithelial cells and human red blood cells (RBCs).

## Materials and Methods

As a first step, we tested commercially synthesized peptides [LL-37, FK13, and FK16 (200 to 0.78 μg/mL)] and antibiotics [gentamicin (64 to 0.5 μg/mL); amikacin (16 to 0.125 μg/mL), and vancomycin (512 to 4 μg/mL)] in broth-microdilution and growth-inhibition assays, in presence or absence of physiological tear salt concentration for elucidation of minimum inhibitory concentration (MIC) and IC_50_ against three virulent strains of *P. aeruginosa*. Bactericidal activity of FK16 was further tested by the SYTOX-green dye uptake assay, using a known membrane-disruptor, melittin as control. Next, the optimum concentration of FK16 and vancomycin was tested for synergism against all PA strains by determining fractional-inhibitory concentration (FIC) index in the presence or absence of physiological tear salt concentration. Gentamicin and amikacin were used as the positive controls. Lastly, we determined the toxicity of FK16 and vancomycin alone or in combination against human corneal epithelial cells (HCE-2) using a cell viability dye assay. The individual methodologies are detailed below.

### Bacterial Strains, Peptides, and Antibiotics

Three *P. aeruginosa* strains were used in this study. PAO1-L (Lausanne sub-line), an invasive strain, was procured from Dr. Stephan Heeb, School of Life Sciences, University of Nottingham, United Kingdom. *P. aeruginosa* ATCC 19660, which is cytotoxic, was obtained commercially from ATCC-LGC Standards, United Kingdom. A clinical isolate of *P. aeruginosa* (PA-OS) from scrapes of human corneal surface with severe corneal melt was obtained from the Department of Clinical Microbiology, Nottingham University Hospitals, United Kingdom. All the work in this study was conducted as per the Health and Safety laboratory guidelines under the Biological safety standards of the University of Nottingham. Cation-adjusted Mueller-Hinton broth (MHB-II), Mueller-Hinton agar (MHA), and Tryptic soy agar (TSA) were purchased from Sigma-Aldrich, United Kingdom. *P. aeruginosa* selective agar F was procured from Merck Millipore, United Kingdom. LL-37 (*LLGDFFRKSKEKIGKEFKRIVQRIKDFLRNLVPRTES*), FK13 (*FKRIVQRIKDFLR*) and FK16 (*FKRIVQRIKDFLRNLV*) were purchased from Anaspec, Fremont, CA, United States. Gentamicin and Amikacin were commercially obtained from Sigma-Aldrich, United Kingdom. Vancomycin Hydrochloride was procured from MP Biomedicals, France.

### Broth Microdilution Assay

Minimum inhibitory concentration was determined for peptides and antibiotics in duplicate using broth microdilution assay according to CLSI guidelines (Wiegand et al., 2008). Twofold serial dilution of peptides and antibiotics were made in duplicate across the 96-well polypropylene microtiter plate using acidified water (0.01% Acetic acid and 0.1% Bovine serum albumin). This resulted in 10 μL of 10x concentration of peptides or antibiotics (gentamicin, amikacin, and vancomycin) in each well. Three to five colonies of PA strain from MHA culture plate were incubated in 5 mL MHB-II in an orbital shaker at 35°C for 16 h. The overnight cultures were sub-inoculated in fresh 5 mL MHB-II at 1 in 50 dilutions and incubated for 3–4 h in the shaking incubator at 35°C until OD_600_ readings gave approximately 2 × 10^8^ CFU/mL. A suspension of 1 × 10^6^ CFU/mL in fresh MHB-II was prepared by further dilution and 90 uL of this suspension was then transferred to each well-containing 10 uL of 10x peptide or antibiotics which resulted in final inoculum of 5 × 10^5^ CFU/mL with desired final concentration of peptide (range 200 to 0.78 μg/mL) or antibiotics [gentamicin (64 to 0.5 μg/mL); amikacin (16 to 0.125 μg/mL), and vancomycin (512 to 4 μg/mL)]. Growth control (only bacteria) and sterility control (no bacteria) were also included in each plate. The plate was incubated at 37°C for 18–21 h and the MIC was determined as the lowest concentration of peptide or antibiotic that inhibited the visible growth of PA strain as observed with the unaided eye.

### Growth Inhibition Assay

Three to five colonies of a PA strain were incubated overnight in 5 mL MHB-II at 35°C in an orbital shaker. The overnight bacterial culture was then diluted to 1 in 50 in fresh MHB-II (5 mL) and further incubated for 3–4 h at 35°C in an orbital shaker to achieve 0.3 OD_600_ (approximately 2 × 10^8^ CFU/mL). Twofold serial dilution of each peptide or antibiotic (gentamicin, amikacin, and vancomycin) in duplicate at 2x concentration in 100 μL was prepared in acidified water containing 0.002% polysorbate-80 (to prevent binding of cationic peptides to the anionic surface of the plate) in a 96-well microtiter polystyrene plate. To each well, 100 μL of bacterial suspension containing 1 × 10^6^ bacteria/mL in MHB-II/0.002% polysorbate-80 was added, which resulted in the inoculum of 5 × 10^5^CFU/mL per well in 200 μL final volume. In addition, individual wells containing vehicle control (1% DMSO) and positive control (50 μg/mL melittin) was also prepared. The plate was incubated at 37°C for 21 h in BMG Clariostar microplate reader and OD_600_ measurements were recorded at 30 min intervals. Two independent experiments were performed for each peptide and antibiotic. Percent growth of bacteria at 13 h for each concentration of peptide and antibiotic was estimated. The IC_50_ value (concentration at which growth reduced to 50%) was then derived from non-linear regression curves using the GraphPad Prism (version 8.0). Growth inhibition assay was also performed for different concentrations of vancomycin (512 to 0 μg/mL, twofold decreasing serial dilutions) against all the three PA strains alone or in combination with FK16 peptide at 0.5x MIC (50 μg/mL) and 0.25x MIC (25 μg/mL), respectively.

### Time-Kill Assay

An overnight culture, each of PAO1-L and PA19660 (approximately 16–18 h) was diluted 50-fold (1 in 50) in fresh MHB-II and further incubated at 35°C in an orbital shaker for 3–4 h until 0.3 OD_600_ (approximately 2 × 10^8^ CFU/mL) was achieved. PAO1-L suspension (2 × 10^6^ CFU/mL) was incubated with FK16 peptide (2 or 0.5x MIC) with or without vancomycin (128 or 256 μg/mL) at 35°C in an orbital shaker. At 0 (starting), 0.5, 3, and 24 h time-points, 10 μL of the mixture was aliquoted and serially diluted in sterile 1x phosphate-buffered saline (PBS), pH 7.2. The diluted bacteria were spread on MHA plates in duplicate and incubated at 37°C for 24 h. Colony count (CFU/mL) was performed and the concentration of FK16 or vancomycin (alone or in combination) was considered bactericidal if there was a ≥ 3 log_10_ reduction in CFU/mL.

### SYTOX-Green Dye Uptake Assay

SYTOX-green does not permeate viable bacteria, but once inside the cytoplasm it binds the nucleic acids and emit fluorescence (λ_*EX*_ = 488 nm; λ_*EM*_ = 520 nm). PAO1 and PA19660 were grown overnight in MHB-II medium and further sub-cultured to a mid-logarithmic growth phase (up to 3 h) at 35°C in an orbital shaker. The suspension was spun at 3000 rpm for 10 min, washed with PBS and diluted to 0.05 OD_600_ in 10% MHB2. To the diluted bacteria, SYTOX-green dye at a final concentration of 2 μM was added and incubated for 15 min. A 96-well black propylene plate containing a mix of 100 μL of bacteria-SYTOX green suspension and 100 μL of FK16 (at 2x concentrations) or melittin (20 μg/mL; positive control) or PBS (negative control) in each well was placed in a fluorescence plate reader (BMG Clariostar) for 30 min. The relative fluorescence unit (RFU) was analyzed by deducting fluorescence from PBS treated bacteria. Two independent experiments were performed in duplicates for each strain.

### Checkerboard Assay

The combined activity and interactions (synergism, sum of activities, or antagonism) between peptides and vancomycin against PA strains in presence or absence of 150 mM NaCl (physiological salt concentration) was determined using checkerboard assay method (7 × 7 matrix format). 25 μL of test peptide at 4x concentration was serially diluted (twofold) in decreasing concentration (final 160 to 0 μg/mL) from column 2 to 8 of a 96-well microtiter polypropylene plate (labeled as ‘Main plate’). In another 96-well plate (labeled as ‘Helper plate’), 25 μL of vancomycin at 4x concentration was prepared in twofold step dilutions (final 512 to 0 μg/mL) from row B to H. The contents from each well of the helper plate was then transferred to the corresponding wells of the main plate to set up an equal 1:1 mix of peptide and vancomycin. The bacterial suspension was prepared (as described in above sections) and diluted to 1 × 10^6^ CFU/mL in fresh MHB-II. 50 μL of diluted suspension (containing 0 or 300 mM NaCl) was then added to each well, resulting in final inoculum of 5 × 10^5^ CFU/mL in 100 μL total volume (final 0 or 150 mM NaCl). Positive control (melittin 50 μg/mL), vehicle control (1% DMSO), and sterility control (no bacteria) was also included in all assay plates. After 21 h of incubation at 37°C, the plates were visually examined for growth. The FIC index for combination of each peptide and vancomycin (in MHB-II ± 150 mM NaCl) against each PA strain was calculated as [(MIC of peptide in combination with vancomycin)/(MIC of peptide alone)] + [(MIC of vancomycin in combination with peptide)/(MIC of vancomycin alone)]. The results were interpreted as FIC ≤ 0.5, synergistic; 0.5 < FIC ≤ 1, additive; 1 < FIC ≤ 4, indifferent; FIC > 4, antagonistic (Ng et al., 2018).

### Cytotoxicity Assay

Human corneal epithelial cells (HCE-2, ATCC) were cultured in keratinocyte serum-free media (KSFM) supplemented with human recombinant epidermal growth factor and bovine pituitary extract. HCE-2 cells were seeded into a 96-well plate at a density of 7.5 × 10^3^ cells per well and allowed to attach overnight. After 24 h of treatment with different concentration of peptides and vancomycin (alone or in combination) in duplicate, the viability of cells was measured using CCK-8 assay kit (Sigma, United Kingdom) as per manufacturer’s guidelines. The WST-8 reagent was bio-reduced by the viable cells into a colored formazan product, which was measured at OD_450_ using BMG Clariostar microplate reader. Percent viable cells was calculated as [(OD_450_ of cells treated with peptide or antibiotic)/(OD_450_ of untreated cells)] x 100.

### Hemolysis Assay

Human blood for hemolysis assay was collected from the healthy subjects with prior consent under the approved ethics (Reference No. 176-1812) from the local Research Ethics Committee of the Faculty of Medicine and Health Sciences, University of Nottingham. Blood was collected in EDTA-coated tube and centrifuged for 10 min at 1300 × *g* for separation of plasma. RBCs were rinsed three times in Ca^2+^/Mg^2+^ free PBS by centrifugation for 5 min at 1300 *g*. RBCs were diluted to 4% vol/vol in PBS and incubated with 100 μL of LL-37 and FK16 (200 to 0.78 μg/mL), vancomycin alone (512 to 2 μg/mL) and in combination with FK16 (50 μg/mL), 1% Triton X-100 (positive control) and PBS (negative control) for 1 h at 37°C in a U-bottom polypropylene 96-well plate. The plate was then centrifuged at 1300 × *g* for 10 min and 100 μL of supernatant was transferred into 96-well polystyrene plate. The absorbance was measured at 540 nm for estimation of percentage hemolysis compared to the positive control. Percent lysis was calculated as [(OD_540_ of suspension from RBC treated with peptide and antibiotic alone or in combination – OD_540_ of suspension from negative control-treated RBCs)/(OD_540_ of suspension treated with positive control – OD_540_ of suspension from negative control-treated RBCs)] x 100.

## Results

### Anti-*Pseudomonas* Activity of Peptides and Antibiotics

LL-37 and its two derivatives, FK13 and FK16, and a range of antibiotics that are commonly used for the treatment of bacterial keratitis were tested against three different strains of *Pseudomonas aeruginosa* (PAO1, PA19660, and PA-OS). The biochemical and structural details of peptides and antibiotics was depicted in the Supplementary Table 1. As shown in Table 1, LL-37 demonstrated higher bactericidal activity against all PA strains when compared to FK16 (50 vs. 100 μg/mL) and FK13 (50 vs. 200 μg/mL), respectively. Of the two shorter peptides, FK16 showed stable bactericidal activity against PAO1, PA19660, and PA-OS. All antibiotics, except vancomycin (>256 μg/mL), showed potent anti-*Pseudomonas* activity. Amikacin, in particular, demonstrated a fourfold higher bactericidal activity compared to gentamicin. However, in the presence of physiological salt concentration (150 mM NaCl), the MIC of gentamicin, amikacin, LL-37, and FK16 was reduced twofold against all tested strains. FK13 was shown to be ineffective at the highest test concentration (200 μg/mL) in the presence of salt against PA19660 and PA-OS indicating that there was no difference in the behavior of FK13 and FK16 with respect to reduction of activity in the presence of salt.

**TABLE 1:** Minimum inhibition concentration (MIC) of the antimicrobial peptides and antibiotics against three different *Pseudomonas aeruginosa* strains.

**μg mL^–1^(μM)**	**PAO1^*a*^**		**PA 19660^*b*^**		**PA-OS^*c*^**	
	
	**NaCl**					
	
	**0 mM**	**150 mM**	**0 mM**	**150 mM**	**0 mM**	**150 mM**
LL-37	50(11.12)	100(22.24)	50(11.12)	100(22.24)	50(11.12)	100(22.24)
FK13	100(28.14)	200(116.28)	200(116.28)	>200(>116.28)	200(116.28)	>200(>116.28)
FK16	100(48.85)	200(97.70)	100(48.85)	200(97.70)	100(48.85)	200(97.70)
Gentamicin	4(8.37)	8(16.74)	4(8.37)	8(16.74)	4(8.37)	8(16.74)
Amikacin	1(1.71)	2(3.42)	1(1.71)	2(3.42)	1(1.71)	2(3.42)
Vancomycin	>256(>176)	>256(>176)	>256(>176)	>256(>176)	>256(>176)	>256(>176)

*^*a*^Opportunistic pathogen that causes invasive infection. ^*b*^Cytotoxic strain from ATCC. ^*c*^Clinical isolate from the ocular surface of patient.*

**FIGURE 1:**
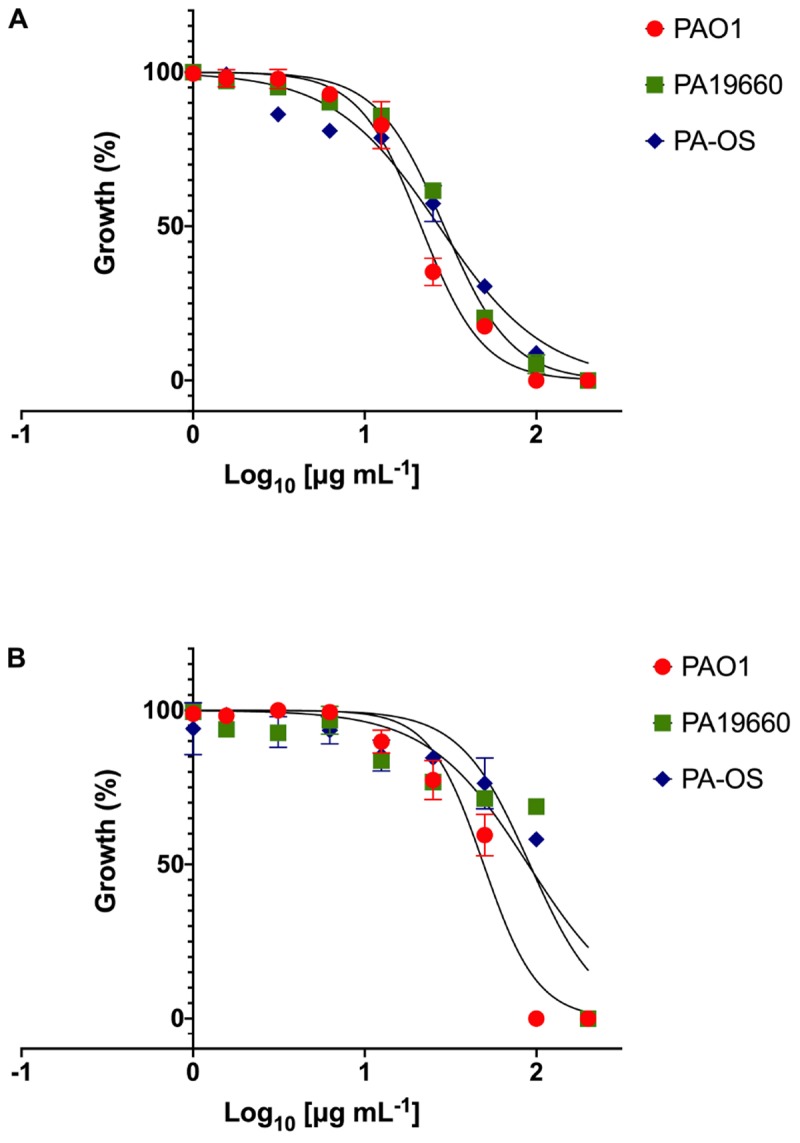
Dose response curves of FK16 **(A)** and FK13 **(B)** against PAO1, PA19660, and PA-OS. Percentage growth is presented as mean ± standard deviation (SD) of two independent experiments performed in triplicate (*n* = 6 data sets). The IC_50_ values were depicted in the Table 2. Some of the error bars are missing due to smaller SD.

As an alternative dynamic methodology to static MIC (Ng et al., 2018), the efficacy of FK16 and FK13 against all PA strains was tested using kinetic growth-inhibition assay. The sigmoidal dose-response curves for FK16 (Figure 1A) showed sharp decrease in percentage growth of PAO1 (IC_50_ = 21.3 ± 3.5 μg/mL), PA19660 (IC_50_ = 29 ± 4.2 μg/mL) and PA-OS (IC_50_ = 27 ± 3.2 μg/mL). In contrast, FK13 (Figure 1B) showed > 2-fold reduced activity compared to FK16 in inhibiting PAO1 (IC_50_ = 49.3 ± 4.2 μg/mL), PA19660 (IC_50_ = 89.5 ± 6 μg/mL), and PA-OS (IC_50_ = 91.5 ± 8 μg/mL). Against PAO1, LL-37 (IC_50_ = 16.3 ± 6.2 μg/mL), gentamicin (IC_50_ = 3 ± 1.5 μg/mL), and amikacin (IC_50_ = 0.5 ± 0.1 μg/mL) all showed higher killing activity (Supplementary Figure 1 and Supplementary Table 2). However, vancomycin required a higher concentration to inhibit PAO1 growth (IC_50_ > 291 μg/mL).

**TABLE 2:** IC_50_ values derived from the dose-response curves for FK13 and FK16.

	**FK13**			**FK16**		
		
	**^∗^ IC_50_(μg mL^–1^)**	**Standard deviation**	***R*^2^**	**^∗^ IC_50_(μg mL^–1^)**	**Standard deviation**	***R*^2^**
PAO1	49.3	2.2	0.965	21.3	3.5	0.990
PA-19660	89.5	6.1	0.814	29	4.1	0.993
PA-OS	91.5	8.1	0.881	27	3.2	0.979

*^∗^IC_50_, concentration at which 50% of the bacterial growth was inhibited.*

**FIGURE 2:**
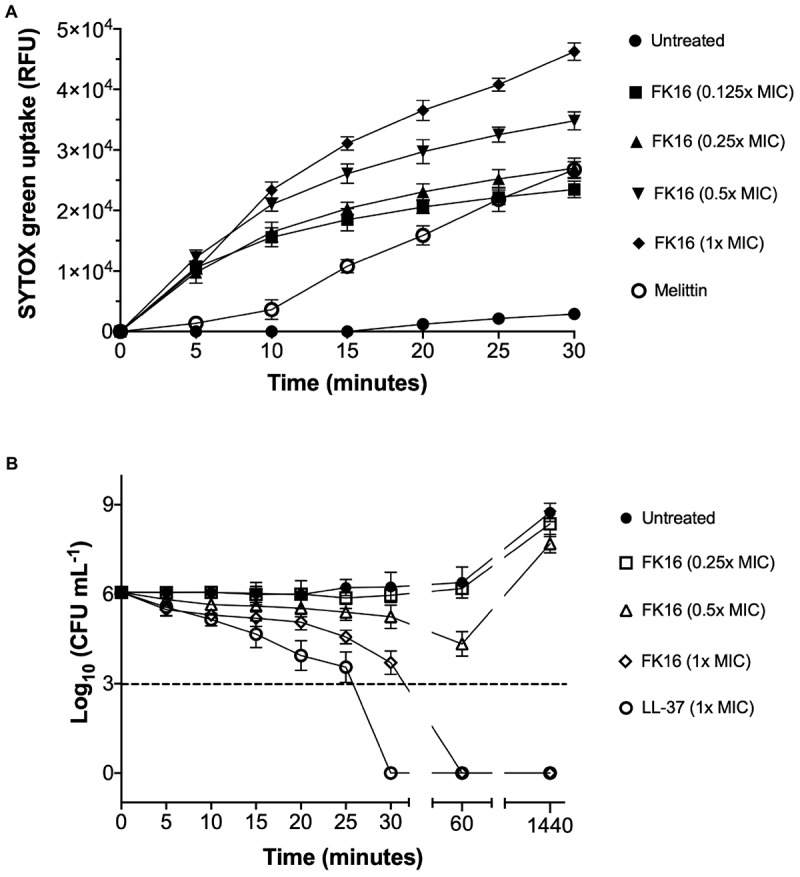
Killing mechanism of FK16 against PAO1. **(A)** Fluorometric analysis of SYTOX-green dye uptake in PAO1. Fluorescence emission was recorded at λ = 520 nm at 5 min interval up to 30 min. Relative fluorescence unit (RFU) vs. time plots for different concentrations of FK16 (in μg/mL), Melittin (20 μg/mL), or PBS control (untreated) were constructed. Data representing three independent experiments performed in duplicate. **(B)** Killing kinetics of PAO1. Bacteria were treated with vehicle (closed circle), FK16 at 1x MIC (open diamond), 0.5x MIC (open triangle) and 0.25x MIC (open square) and LL-37 at 1x MIC (open circle) for 5, 10, 15, 20, 25, 30, 60, and 1440 min, respectively. ‘0 h’ represents the starting inoculum. Colonies were counted (CFU/mL) 24 h post-treatment from serial dilutions in duplicate and presented in a logarithmic scale. Data is presented as mean ± standard deviation (SD) of three independent experiments. Some of the error bars are missing due to smaller SD.

GF-17 (also known as N-glycinated FK16) has been previously shown to kill *Escherichia coli* via membrane-disruption (Wang et al., 2017). To validate whether the FK16 also kills *P. aeruginosa* utilizing a similar mechanism, we performed the SYTOX-green dye uptake assay. The increased intensity of fluorescence corresponds to the membrane disruption and binding of the dye to the nucleic acids of the bacterium. As shown in Figure 2A, the influx of SYTOX-green dye in PAO1 was directly proportional to the concentration of FK16 and the maximum relative fluorescence was noted at 1x MIC. Melittin, a known membrane disruptor from honey-bee venom, was used as a positive control. We further assessed the killing efficacy of FK16 (1, 0.5, and 0.25x MIC) against PAO1 at 5-min interval up to 30 min and then at 60 min and 24 h, respectively. As shown in Figure 2B, FK16 at 1x MIC was shown to kill PAO1 at 60 min with no growth noted up to 24 h. Whilst LL-37 at 1x MIC was shown to kill PAO1 at 30 min.

### Antimicrobial Effect of Peptides and Vancomycin Combination Against *P. aeruginosa*

As demonstrated above, vancomycin failed to exhibit significant bactericidal activity against *P. aeruginosa* (MIC > 256 and IC_50_ > 291 μg/mL). This is due to poor permeation of vancomycin through the outer membrane of *P. aeruginosa*. Based on the ability of FK16 to modulate membrane permeability, we hypothesized that in combination with FK16 the antimicrobial susceptibility of vancomycin against *P. aeruginosa* could be augmented. To test this hypothesis, we performed the growth-inhibition assay for the combination of FK16 (at sub-MIC levels; 25 and 50 μg/mL) and vancomycin (512 to 0 μg/mL in twofold serial dilution) against all three PA strains. Kinetic kill curves (Supplementary Figure 2) were plotted using OD_600_ readings that were recorded at 30 min intervals up to 21 h. The normalized percentage growth of each strain at 13- and 21-h time point was then calculated for analysis of FK16 + vancomycin combination against *P. aeruginosa*.

**FIGURE 3:**
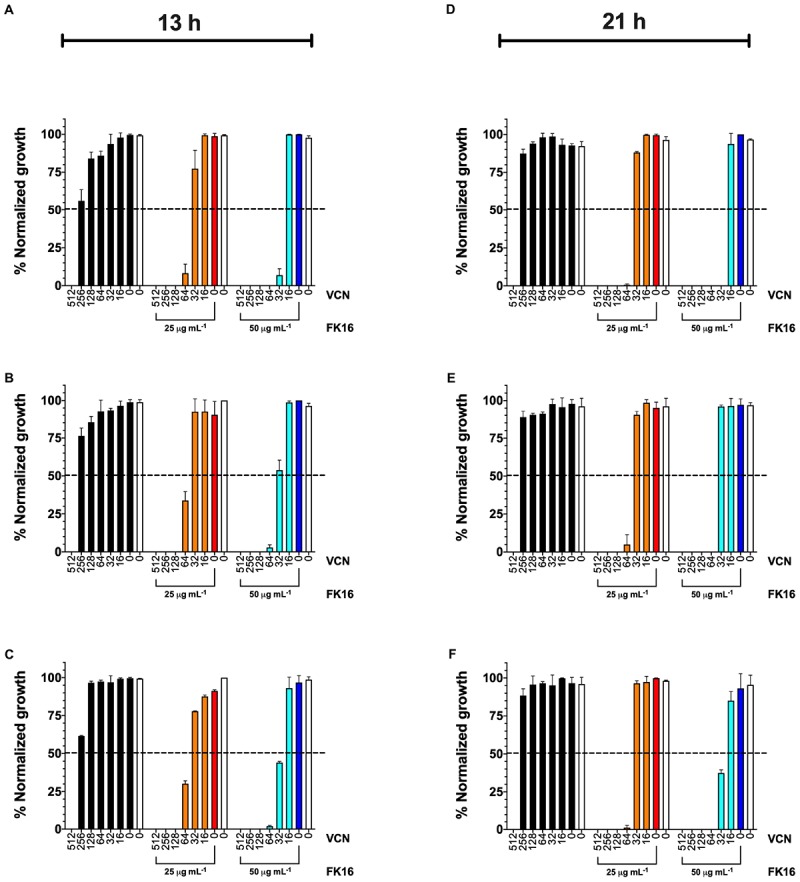
Normalized percentage growth of PAO1 **(A,D)**, PA-19660 **(B,E),** and PA-OS **(C,F)** from OD600 recordings at 13-h (early stationary phase) and 21-h (late stationary phase) time point following treatment with FK16 and vancomycin combination. Vancomycin (VCN) alone treatment (512 to 0 μg/mL) is represented with black bars. VCN (512 to 0 μg/mL) + FK16 at 25 μg/mL (orange bars) and VCN (512 to 0 μg/mL) + FK16 at 50 μg/mL (cyan bars). Vehicle treatment (clear bar) and FK16 alone at 25 μg/mL (red bar) and 50 μg/mL (blue bar). Data is presented as mean ± standard deviation (SD) of two independent experiments performed in triplicate (*n* = 6 data sets). Some of the error bars are missing due to smaller SD. Inhibition is seen up to the 21-h time point **(D–F)**.

As shown in Figure 3 (orange bars), at 21-h time point, FK16 (25 μg/mL) enhanced the killing efficacy of vancomycin by eightfold (512 vs. 64 μg/mL) against PAO1 (Figure 3D), PA-19660 (Figure 3E), and PA-OS (Figure 3F). Moreover, at 25 μg/mL FK16 + 64 μg/mL vancomycin combination, >99.5% normalized growth of PAO1 (Figure 3D) and >95% of both PA19660 (Figure 3E) and PA-OS (Figure 3F) were shown to be inhibited. To assess whether the bactericidal activity of vancomycin is dependent on FK16 concentration, we increased the concentration of FK16 to 50 μg/mL (0.5x MIC) in the growth inhibition assay. We have noted an 16-fold improvement in bactericidal activity against PAO1 [512 vs. 32 μg/mL; (Figure 3D, cyan bars)] and eightfold improvement against PA19660 and PA-OS (512 vs. 64 μg/mL; Figures 3E,F, cyan bars), respectively.

**FIGURE 4:**
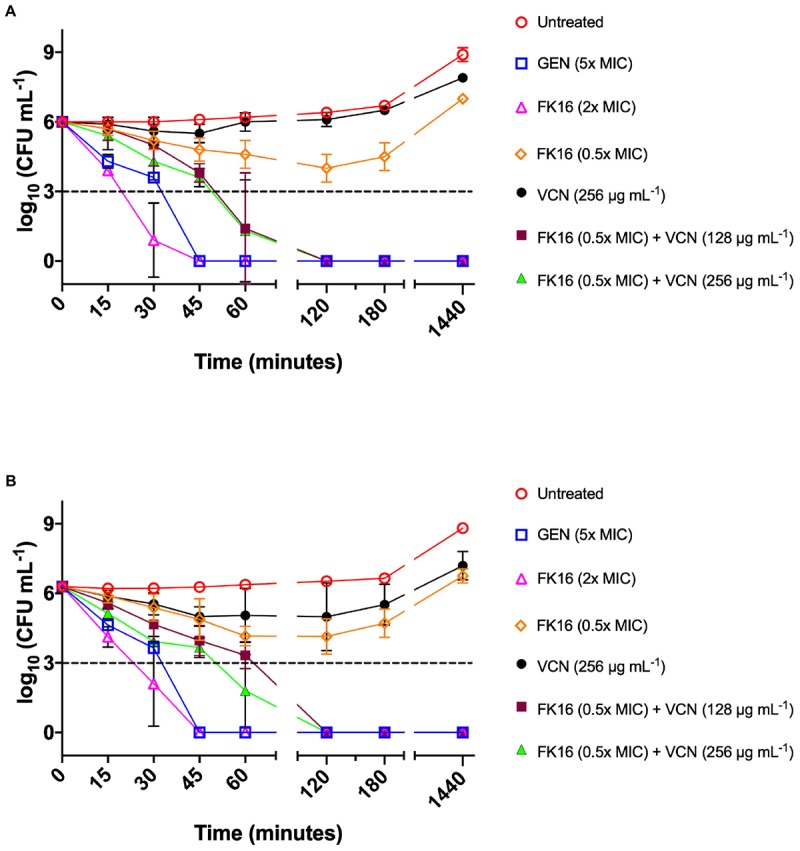
Time-kill curves of FK16 and vancomycin alone or in combination against PAO1 **(A)** and PA19660 **(B)**. Bacteria were treated with FK16 alone (2x MIC; open magenta triangle) and (0.5x MIC; open orange diamond), VCN alone (256 μg/mL; closed black circle), FK16 (0.5x MIC) + vancomycin (VCN; 128 μg/mL closed brown square), FK16 (0.5x MIC) + VCN (256 μg/mL; closed green triangle), Gentamicin (5x MIC; open blue square) and vehicle (open red circle) for 15, 30, 45, 60, 120, 180 and 1440 min, respectively. ‘0 minutes’ represents the starting inoculum. Colonies were counted (CFU/mL) 24 h post-treatment from serial dilutions in duplicate and presented in a logarithmic scale. Data is presented as mean ± standard deviation (SD) of three independent experiments. Some of the error bars are missing due to smaller SD.

We further validated the killing kinetics of FK16-vancomycin combination against PAO1 and PA19660 in a time-kill assay. Gentamicin, which was used as a positive control (5x MIC; 20 μg/mL) and FK16 at twofold above the MIC (200 μg/mL) have been shown to completely kill PAO1 (Figure 4A) and PA19660 (Figure 4B) at 45 min. Notably, FK16 (50 μg/mL; sub-MIC) in combination with vancomycin (128 μg/mL) have been shown to completely inhibit the growth of both strains at 2 h and demonstrated sustained activity up to 24 h. With combination of FK16 (50 μg/mL; sub-MIC) and vancomycin (256 μg/mL; twofold higher concentration), PAO1 (Figure 4A) and PA19660 (Figure 4B) were shown to be reduced > 3 log_10_(CFU/mL) within 60 min and complete kill achieved by 2 h with no growth noted up to 24 h.

To establish a synergistic or additive effect of the FK16 and vancomycin combination, a MIC-based checkerboard assay against all three PA strains was performed. In addition, we have also examined the combination effect in the presence of 150 mM NaCl. As depicted in Table 3, FK16-vancomycin combination has demonstrated synergistic bactericidal activity against PAO1 (FICI = 0.25), PA19660 (FICI = 0.375), and PA-OS (FICI = 0.375), respectively. In the presence of physiological salt, combination of FK16 and vancomycin remained synergistic against PAO1 (FICI = 0.375). Although the combination effect against PA19660 (FICI = 0.50) and PA-OS (FICI = 0.50) have been slightly reduced in assay buffer containing 150 mM NaCl, the FIC indices against both strains were still at the borderline between synergism and additive level.

**TABLE 3:** Effect of FK16 and Vancomycin combination in presence and absence of physiological salt concentration.

***FK16* + *VCN***	***0 mM NaCl***		***150 mM NaCl***	
		
	**FIC index**	**Interpretation*^a^***	**FIC index**	**Interpretation*^a^***
PAO1	0.25	Synergy	0.375	Synergy
PA-19660	0.375	Synergy	0.50	Synergy
PA-OS	0.375	Synergy	0.50	Synergy

*^*a*^FIC Index = (MIC FK16 _*comb*__/_MIC FK16 _*alone*_) + (MIC VCN _*comb*__/_MIC VCN _*alone*_). FIC ≤ 0.5 represents synergy. FIC between 0.5 and 2 represents additive effect. FIC ≥ 2 represents inhibitory effect.*

### Potential Toxicity of Peptides and Vancomycin Toward Human Corneal Epithelial Cells and Human Red Blood Cells

**FIGURE 5:**
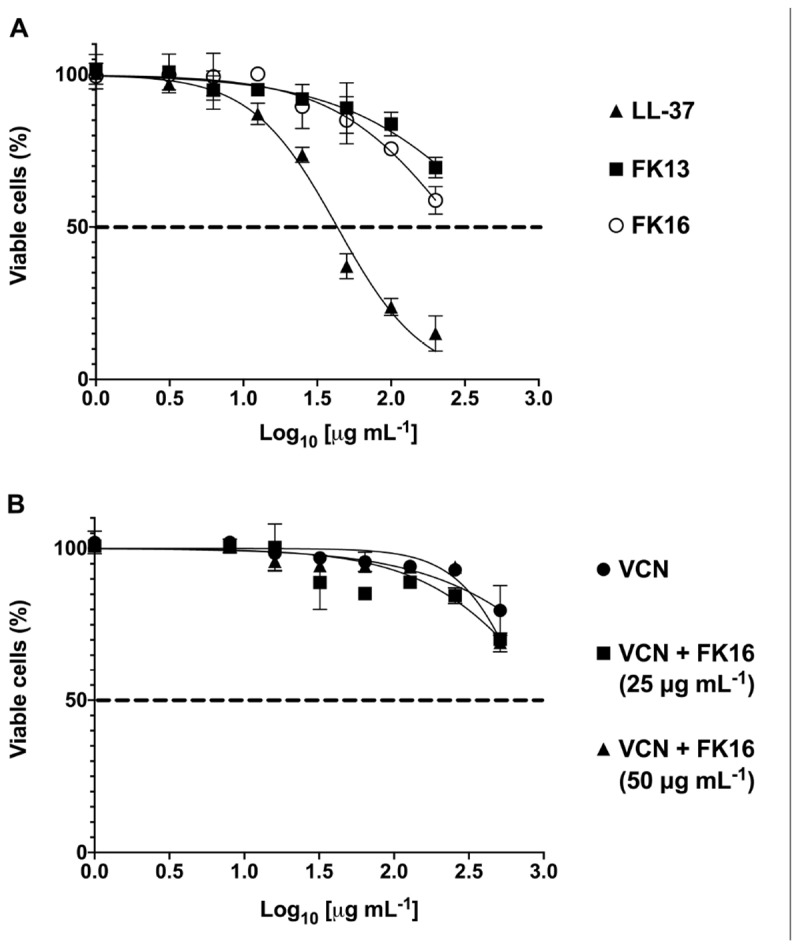
Toxic effects of peptides **(A)** and vancomycin (VCN) + FK16 combination **(B)** against human corneal epithelial cells (HCE-2). After 24 h of treatment with different concentration of peptides alone or in combination with different concentration of VCN, HCE2 viability was assessed with CCK8 reagent. EC_50_ and V_*max*_ was derived (Table 4) from the percentage viable cell curves using GraphPad Prism (ver. 8.0). Data is presented as mean ± standard deviation (SD) of three independent experiments performed in duplicate. Some of the error bars are missing due to smaller SD.

Antimicrobial peptides at higher concentrations have been shown to elicit toxic responses on host tissue. This non-selective effect of AMPs has therefore limited their clinical application (Haney et al., 2019). Here, we evaluated the potential cytotoxic effects of cationic peptides (LL-37, FK13, and FK16), vancomycin and different concentrations of FK16 in combination with vancomycin on human corneal epithelial cells (HCE-2, ATCC) until 24 h treatment duration. As shown in Figure 5A, LL-37 has demonstrated significant cytotoxic effects on HCE2 (EC_50_ = 43.20 ± 4.08 μg/mL). Notably, EC_50_ value of LL37 was matching to its 1x MIC levels against *P. aeruginosa* (Table 1). FK13 and FK16 were shown to be non-toxic to HCE2 (EC_50_ > 200 μg/mL; Table 4). Similarly, treatment of HCE2 for 24 h with different concentrations of vancomycin either alone (EC_50_ > 512 μg/mL) or in combination with FK16 at 25 μg/mL (EC_50_ > 512 μg/mL) or 50 μg/mL (EC_50_ > 512 μg/mL) have not elicited toxic effects (Figure 5B).

**TABLE 4:** Toxic effects of peptides and vancomycin against HCE-2 cells.

	**^a^EC_50_μg mL^–1^ (SD)**	**^b^V_max_% (SD)**
LL-37	43.20 (4.08)	15.08 (5.76)
FK13	>200	69.49 (3.36)
FK16	>200	58.75 (4.54)
VCN	>512	79.67 (8.15)
VCN + FK16 (25 μg mL**^–^**^1^)	>512	70.21 (2.08)
VCN + FK16 (50 μg mL**^–^**^1^)	>512	69.18 (3.09)

*^*a*^EC_50_ – Concentration at which 50% of the cell viability was reduced. ^*b*^V_*max*_ – Percentage (%) cell viability at the highest concentration of peptides and vancomycin (all peptides = 200 μg mL**^–^**^1^ and vancomycin = 512 μg mL**^–^**^1^).*

**FIGURE 6:**
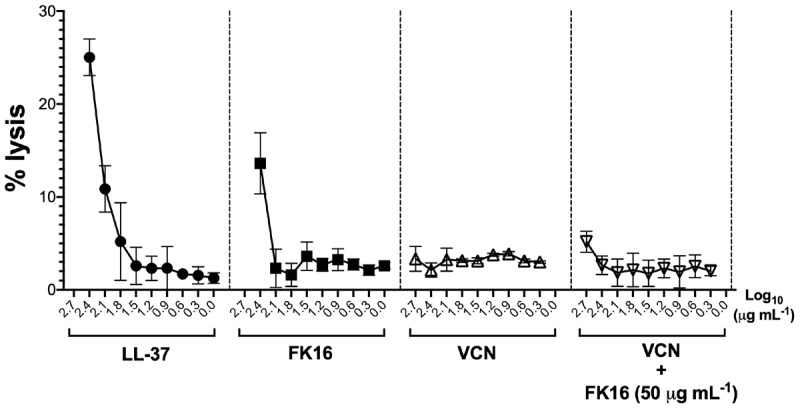
Lytic effect against human red blood cells (RBCs). After 1 h of treatment with different concentration of LL-37 (closed circle; 200 to 0 μg/mL), FK16 alone (closed square; 200 to 0 μg/mL), VCN alone (upright open triangle; 512 to 8 μg/mL) and in combination with FK16 at 50 μg/mL (inverted open triangle), hemolysis was assessed by measuring optical absorbance of supernatant at 540 nm. Percentage lysis curves were plotted using GraphPad Prism (ver. 8.0). Data is presented as mean ± standard deviation (SD) of three independent experiments performed in duplicate. Some of the error bars are missing due to smaller SD.

We also assessed the hemolytic effect of peptides and vancomycin alone or in combination on human red blood cells (hRBC). LL-37 displayed 25.03 ± 1.97 percent lysis of hRBC at 256 μg/mL (Figure 6). Whereas FK16 exhibited 13.61 ± 3.29% lysis at 256 μg/mL. Vancomycin alone (512 to 2 μg/mL) or in combination with FK16 (50 μg/mL) did not induce significant hemolysis.

## Discussion

The growing threat of MDR *P. aeruginosa* has profoundly affected a diverse patient cohort and health services worldwide. A key strategy to counter the antimicrobial resistance would be to exploit the potential of AMPs to improve the effectiveness of conventional antibiotics (Hollmann et al., 2018). This approach was deemed unique because of the differences in the mechanisms of action of AMPs compared to antibiotics.

In the present work, we have selected LL-37 and its shorter peptides on the basis of previous findings from our and another laboratories (McIntosh et al., 2005; Huang et al., 2006, 2007; Wang et al., 2012, 2014; Rajasekaran et al., 2017). LL-37 was shown to be abundantly expressed on the human ocular surface in response to *P. aeruginosa* (McIntosh et al., 2005; Huang et al., 2006). Moreover, its genetic deletion in mouse has been shown to increase the susceptibility to *Pseudomonas* keratitis (Huang et al., 2007). Thus, there is sufficient evidence in the literature to justify a study to explore the efficacy and safety of AMPs or AMP-derived peptides in isolation and in combination with antibiotics.

Published results from the Steroids for Corneal Ulcers Trial (SCUT) have shown that the corneal ulcers with genotypically invasive *P. aeruginosa* subgroup have marked differences in the clinical presentation and responses to treatment when compared to genotypically cytotoxic *P. aeruginosa* subgroup (Borkar et al., 2013). A subsequent report from the SCUT study has further demonstrated that exoU(+) encoding cytotoxic *P. aeruginosa* isolates were significantly resistant to ciprofloxacin, gatifloxacin, and ofloxacin compared to exoU(−) *P. aeruginosa* strain (Borkar et al., 2014). This illustrates that the specific virulence determinants of a single species of a pathogen respond differently to treatment. Although LL-37 was found to be twofold more potent than its shorter peptides against *P. aeruginosa*, its detrimental effect on corneal epithelial cells made it undesirable for further synergism experiments in the context of ocular surface infections. Our results agree with earlier studies which also demonstrated the toxicity of LL-37 against RBCs and a variety of human cell lines (Jaskiewicz et al., 2018; Haney et al., 2019).

Structure-activity relationship (SAR) studies have enabled the development of short fragments of LL-37 with improved cell selectivity (Mookherjee and Hancock, 2007; Mishra et al., 2013; Wang et al., 2014). It was shown that the region between 2 and 31 residues of LL-37 is important for antibacterial activity (Li et al., 2006). A recent study utilizing nuclear magnetic resonance (NMR) has confirmed that the amino acid residues between 17 and 32 (i.e., FK16) have strong binding affinity toward anionic (bacterial) but not zwitterionic (host) model membranes (Wang et al., 2012). Moreover, FK16 has been shown to exhibit strong bactericidal activity against ESKAPE organisms (*Enterococcus faecium*, *Staphylococcus aureus*, *Klebsiella pneumoniae*, *Acinetobacter baumanii*, *Pseudomonas aeruginosa*, and *Enterobacter* spp.) (Mishra and Wang, 2017). GF17, a N-glycinated variant of FK16, has also been shown to exhibit anti-cancer (Ren et al., 2013), anti-bacterial (Wang et al., 2012; Kiattiburut et al., 2018) and anti-viral activities (He et al., 2018). It was further confirmed that GF17 and its structurally modified variants were able to kill a variety of Gram-negative and Gram-positive bacteria *via* membrane disruption (Wang et al., 2017). In addition, GF17 and 17BIPHE2 (second generation peptide based on GF17 structure) precoated biomaterials were shown to prevent bacterial biofilm formation both in *in vitro* and *in vivo* model systems (Mishra and Wang, 2017). Consistent with the above reports, we have also demonstrated that FK16 is more effective than FK13 against all *P. aeruginosa* strains and specifically damages the cell membranes as confirmed with the SYTOX-green uptake assay. However, the activity of FK16 was found to be slightly reduced in the presence of physiological salt conditions. A similar observation has been reported with GF17 activity against *E. coli* (Wang et al., 2017), which suggested that the rate of interaction between the peptide and bacterial membrane may reduce under physiological salt conditions.

Although antibiotics have been shown in this study to kill *P. aeruginosa* at lower concentrations than LL-37 and its smaller peptides, they are clinically used at very high concentration, i.e., between 0.3 and 1.5% as topical formulations (3 mg/mL; used at > 1500x MIC) for the treatment of *Pseudomonas* keratitis (Dua et al., 2012; Otri et al., 2013). AMPs, on the other hand, have been shown to exhibit *in vivo* bactericidal efficacy at low micromolar concentrations (Gordon et al., 2005; Beaumont et al., 2014; Kolar et al., 2015; Pletzer et al., 2017; Wuerth et al., 2019). Given the low antimicrobial efficacy of the small peptides, which is compounded by the tear salt concentration and presence of other proteases, a 4x MIC concentration (800 μM) would be needed for therapeutic effect. This would be too high but in combination with antibiotics, the former can be used in sub-inhibitory concentrations thus minimizing their cytotoxic effects whilst potentiating the effect of the antibiotics to effectively treat ocular surface infections.

The standard MIC assay is useful for ascertaining the antibacterial efficacy of a test compound at the end of a fixed time-point (18 or 21 h). However, it does not provide detailed information on the growth-rate inhibition of bacteria in response to different concentrations of an antimicrobial agent (Abdelbaqi et al., 2016; Ng et al., 2018). Here, we further validated the antimicrobial activity of our peptides and antibiotics utilizing a dynamic pharmacological approach for the generation of dose-response curves. The resultant growth inhibition curves from OD_600_ measurements have allowed us to report the IC_50_ readings (concentration at which 50% of the bacterial growth was inhibited) in addition to MIC values for all our test compounds. In our experiments ‘growth inhibition’ could be a reflection of the initial kill of the bacteria by the agents used rather than retardation of growth (of the surviving bacteria) as would be seen by alteration of pH for example. However, the use of term ‘inhibition’ here would be in the same context as used in MIC assay where the ‘growth inhibition’ is a reflection of the killing of bacteria.

We have noted that the activity of FK13 but not FK16 was greatly reduced against cytotoxic PA19660 and ocular PA isolate. Although not tested here, it is likely that FK13 may require higher concentration (>200 μg/mL) to achieve similar efficacy as FK16 against *P. aeruginosa* in the presence of salt. Moreover, the enhanced activity of FK16 could be attributed to the additional hydrophobic residues on the C-terminus (Leu_31_ and Val_32_ as per LL-37 sequence). Interestingly, a previous structural study has provided evidence that C-terminus residues of FK16 were shown to form a 3_10_ helix (Li et al., 2006). However, implication of these hydrophobic residues to the functional activity of FK16 against Gram-negative strains of different virulence was not well-understood. Our comparison of FK13 and FK16 activity have revealed that the hydrophobic C-terminus of FK16 is key for stable bactericidal activity against cytotoxic and ocular *P. aeruginosa* strains. It is anticipated that further SAR studies would validate our findings and may lead to the development of rationally designed analogs of FK16.

The results from our study are in agreement with earlier reports that vancomycin exhibits much weaker activity against *P. aeruginosa* (Alvarez et al., 2016; Ng et al., 2018; Pletzer et al., 2018). Earlier studies have demonstrated that polymyxins and ciprofloxacin are capable of enhancing the activity of vancomycin against *P. aeruginosa* (Day et al., 1993; Ng et al., 2018). However, the wider use of antibiotic combinations has been greatly discouraged due to the increased risk of toxicity (Poole, 2011; Dua et al., 2012; Vazirani et al., 2015).

Synergism between antibiotics and AMPs has been demonstrated both in *in vitro* and *in vivo* infection model systems (Blazewicz et al., 2018; Pletzer et al., 2018). Synthetic AMPs such as 1008 and DJK-5 were shown to kill *P. aeruginosa* via disruption of stringent-stress response pathway (Pletzer et al., 2017). It was further demonstrated that the cutaneous abscesses caused by ESKAPE pathogens can be successfully treated with the combination of peptides and conventional antibiotics (Pletzer et al., 2018). A modified FK13 peptide, FK13-a1, was also shown to enhance the activity of chloramphenicol against MDR bacteria both in the presence and absence of salt (Rajasekaran et al., 2017).

Our results have demonstrated that FK16 at sub-MIC levels was capable of enhancing the susceptibility of *P. aeruginosa* against vancomycin (up to eightfold). Moreover, further analysis in checkerboard assays has shown that FK16 activity in combination with vancomycin is stable in the physiological tear salt conditions. Although MIC-based checkerboard assay has its own limitation, our results from plate-count experiments implicated a possible synergism between FK16 and vancomycin against both invasive and cytotoxic strain of PA. Thus, while further assessment of FK16-vancomycin in *in vivo* bacterial keratitis model is required, it may be reasonable to infer that the observed synergism/additive effect would likely be effective in the treatment of *P. aeruginosa* that have already demonstrated multi-drug resistance. The exact mechanism of the enhanced effect of the two agents together is unclear. Though increased permeabilization of the membrane(s) is observed with FK16, it does not directly suggest the mechanism by which the enhanced effect is achieved. Despite meticulous attention to details regarding time of incubation, volume and concentration of inoculum, subtle errors can inadvertently creep in and affect the outcome and consequent FIC index. Thus, though suggestive of synergism, it is not conclusive. To bring this one step closer to the therapeutic realization, we have also shown that FK16 + vancomycin combination is non-toxic to the human corneal epithelial cells and human RBCs. Therefore, our *in vitro* results have the potential to form the basis for early preclinical studies particularly for the assessment of toxicity and pharmacokinetic properties of combination therapy including compatibility of formulation, stability, and effective administration route.

In summary, FK16 alone or in combination with vancomycin has shown enhanced ability to kill *P. aeruginosa* of different virulence without eliciting host cell toxicity. These results provide further credence to the overarching concept of developing the next generation of AMP-antibiotic combination therapies as a viable option to counter antibiotic resistance. Future studies to test the preclinical efficacy of FK16-vancomycin synergism against PA in an *in vivo* model of bacterial keratitis are planned to take this concept further toward clinical realization.

## Data Availability Statement

All datasets generated for this study are included in the manuscript/Supplementary Files.

## Ethics Statement

Human blood for hemolysis assay was collected from the healthy subjects with prior consent under the approved ethics (Reference No. 176-1812) from the local Research Ethics Committee of the Faculty of Medicine and Health Sciences, University of Nottingham.

## Author Contributions

IM and HD conceived and designed the experiments. IM performed the experiments and prepared the figures. DS, MN, and LM contributed reagents, materials, analysis tools, and proofread and approved the final draft. IM and HD analyzed the data and prepared the draft of manuscript.

## Conflict of Interest

HD is consultant to Dompe, Santen, Thea, and Visufarma and holds shares in Glaxosmithkline and NuVision biotherapeutics. The remaining authors declare that the research was conducted in the absence of any commercial or financial relationships that could be construed as a potential conflict of interest.
