# Supplementary figures and images for: Cathelicidin-Derived Synthetic Peptide Improves Therapeutic Potential of Vancomycin Against Pseudomonas aeruginosa
Source: Front Microbiol. 2019 Sep 19;10:2190. doi: 10.3389/fmicb.2019.02190 (PMC6761703; doi:10.3389/fmicb.2019.02190)

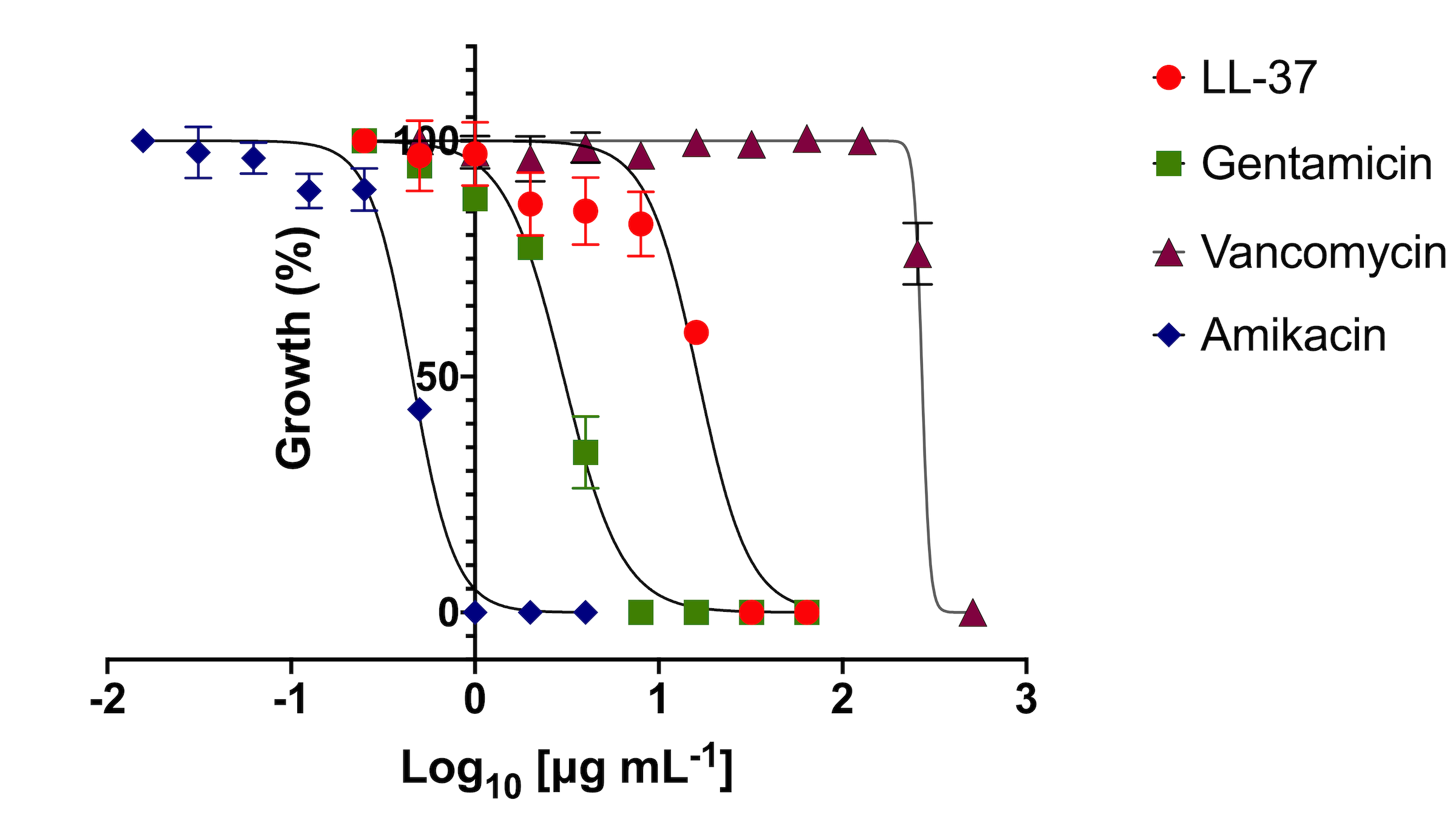

Supplement: Supplementary file 1 [file Image_1.TIFF]

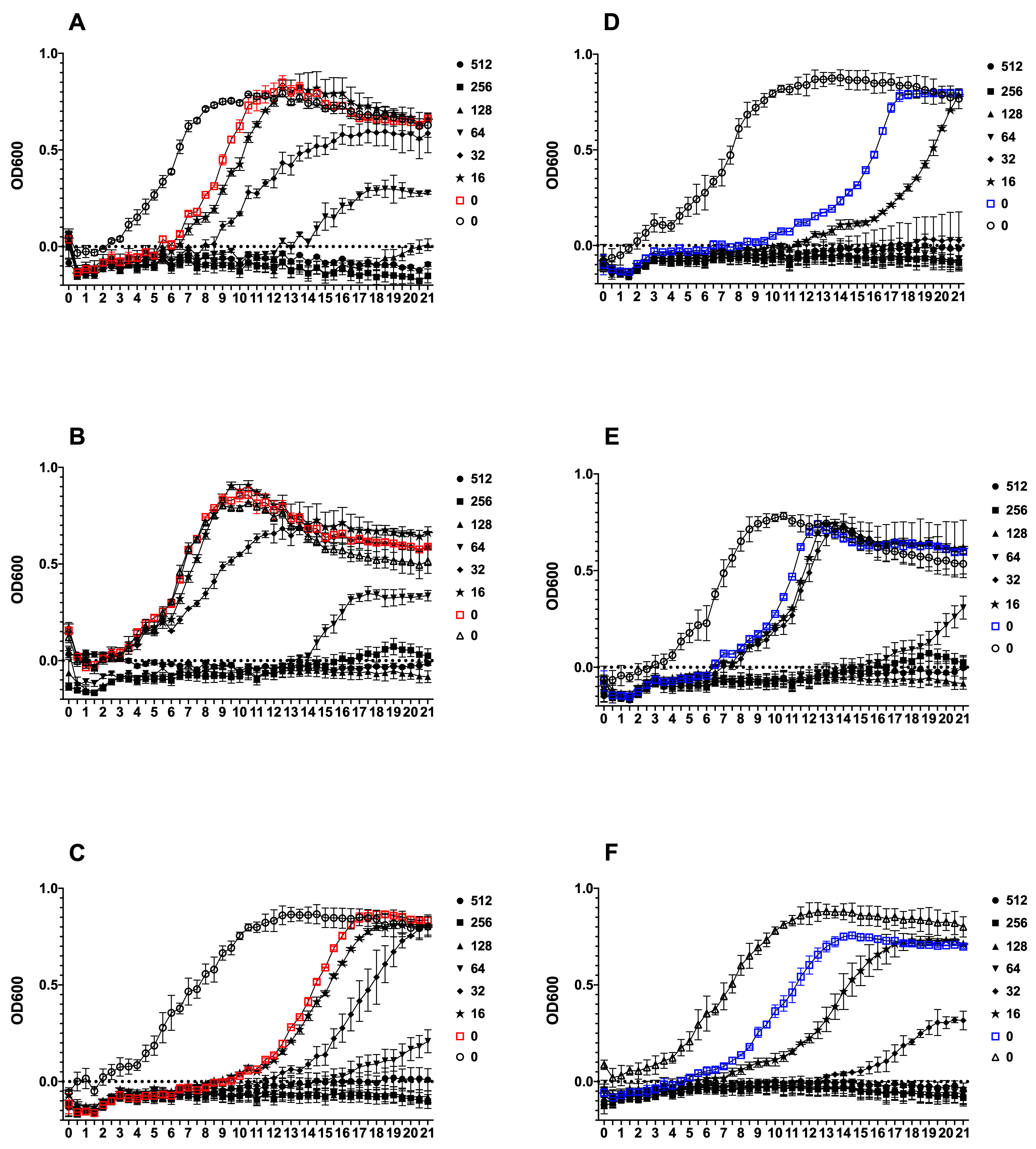

Supplement: Supplementary file 2 [file Image_2.TIFF]
