# Supplementary material for: Cathelicidin-Derived Synthetic Peptide Improves Therapeutic Potential of Vancomycin Against Pseudomonas aeruginosa
Source: Front Microbiol. 2019 Sep 19;10:2190. doi: 10.3389/fmicb.2019.02190 (PMC6761703; doi:10.3389/fmicb.2019.02190)
Supplement: Supplementary file 3 [file Table_1.DOCX]

**Supplementary Material**

*Supplementary Table 1. Details of peptides and antibiotics used in this study*

| **Hydrophobicity** | 47.79 | 35.69 | 40.37 | - | - | - |
| --- | --- | --- | --- | --- | --- | --- |
| **Net charge at**  **pH 7.0** | +6 | +4 | +4 | - | - | - |
| **Molecular weight**  **(g/mol)** | 4493.26 | 1719.09 | 2045.48 | 1449.3 | 477.6 | 585.6 |
| **Amino acid sequence and chemical formula**  *(No modifications at N and C-terminus)* | *LLGDFFRKSKEKIGKEFKRIVQRIKDFLRNLVPRTES* | *FKRIVQRIKDFLR* | *FKRIVQRIKDFLRNLV* | *C_66_H_75_Cl_2_N_9_O_24_* | *C_21_H_43_N_5_O_7_* | *C_22_H_43_N_5_O_13_* |
| **Peptide** | LL-37 | FK13 | FK16 | Vancomycin | Gentamicin | Amikacin |

*Supplementary Table 2. IC_50_ values for LL-37 and antibiotics*

|  | **IC_50_**  **(μg mL^-1^)** | **Standard deviation** | **R^2^** |
| --- | --- | --- | --- |
| **LL-37** | 16.3 | 6.2 | 0.942 |
| **Gentamicin** | 3 | 1.5 | 0.990 |
| **Amikacin** | 0.5 | 0.1 | 0.989 |
| **Vancomycin** | > 291 | - | 0.990 |


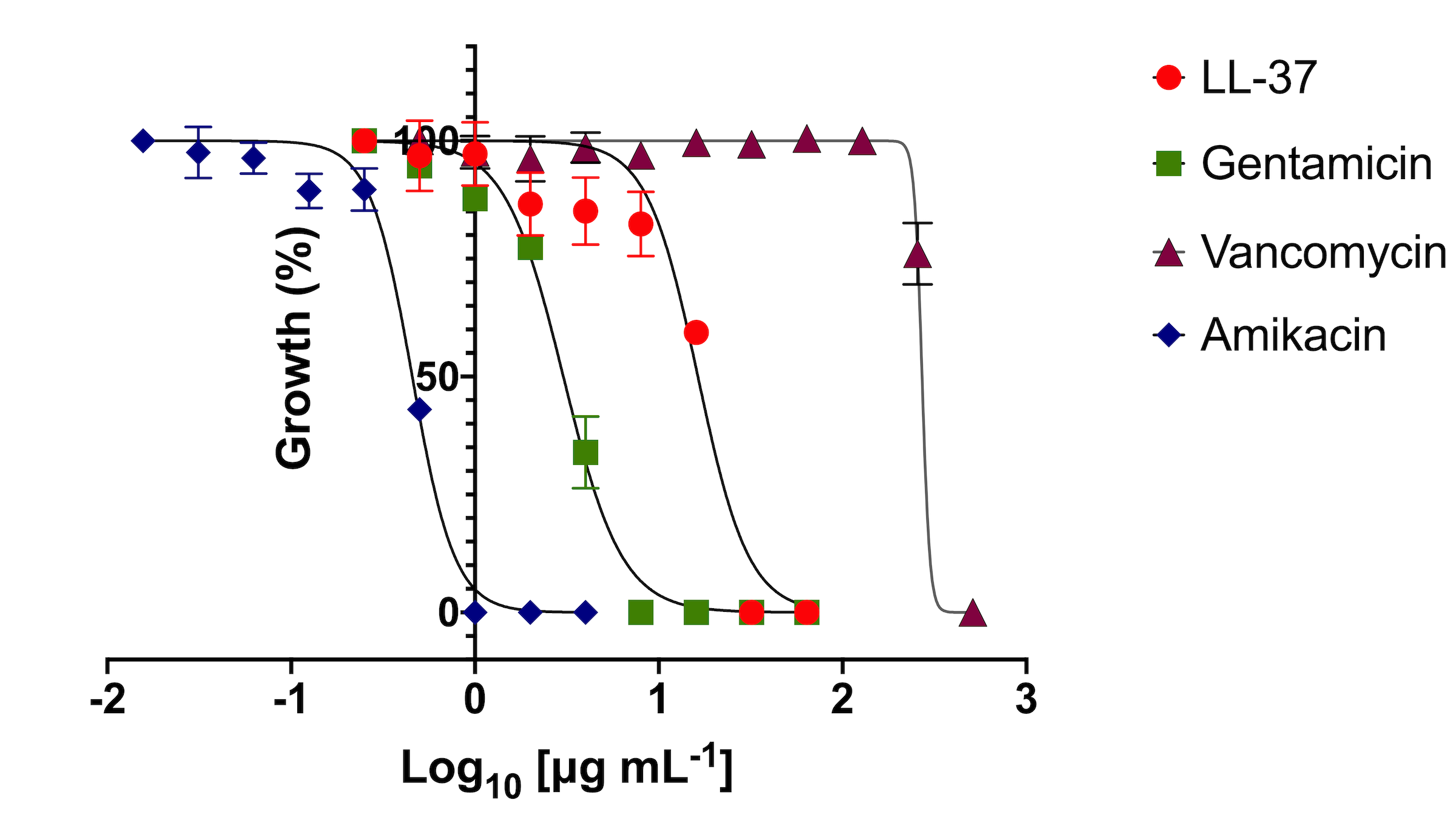


**Supplementary figure 1.** Dose response curve of LL-37, gentamicin, vancomycin and amikacin against PAO1. Data represents mean ± standard deviation (SD) of two independent experiments performed in triplicate. The IC_50_ values were depicted in the supplementary table 1. Note: Some of the error bars are missing due to smaller SD.


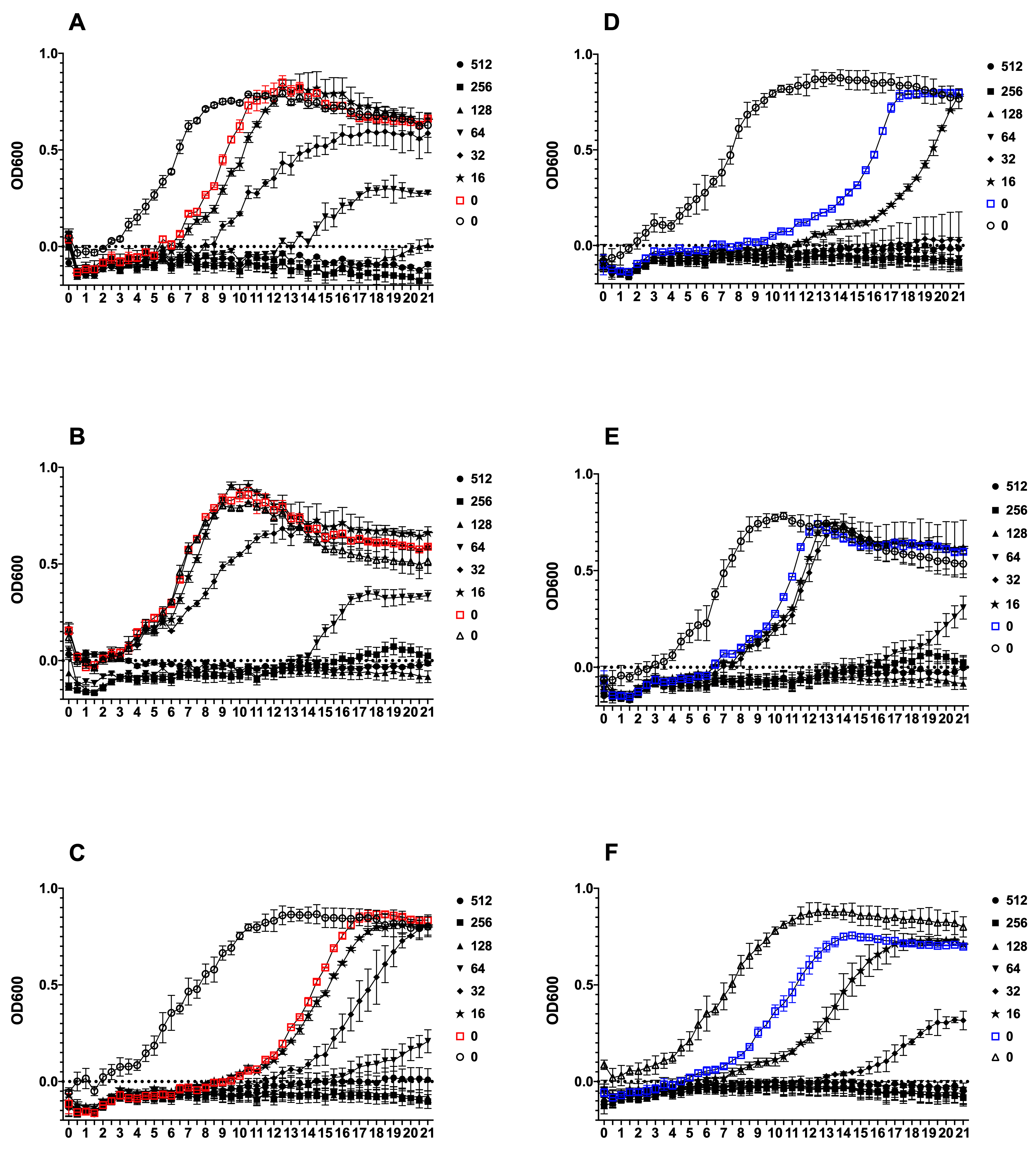


**Supplementary figure 2.** Kinetic kill curves of FK16 and vancomycin combination. Optical density (OD) at λ=600nm were recorded at 30 minutes interval up to 21 hours. OD600 vs time plots for different concentrations of vancomycin in combination with FK16 at 25 μg/mL (A to C) or FK16 at 50 μg/mL (D to F) were constructed. FK16 alone at 25 μg/mL (open red squares) and at 50 μg/mL (open blue squares). A and D represents PAO1; B and E represents PA-19660; and C and F represents PA-OS. Data represents mean ± standard deviation (SD) of two independent experiments performed in triplicate. The normalised percentage growth at 21-hour (end-point) has been shown in figure 2. Note: Some of the error bars are missing due to smaller SD.
